# Melatonin Maintains Inner Blood–Retinal Barrier by Regulating Microglia *via* Inhibition of PI3K/Akt/Stat3/NF-κB Signaling Pathways in Experimental Diabetic Retinopathy

**DOI:** 10.3389/fimmu.2022.831660

**Published:** 2022-03-15

**Authors:** Lei Tang, Chaoyang Zhang, Lixia Lu, Haibin Tian, Kun Liu, Dawei Luo, Qinghua Qiu, Guo-Tong Xu, Jingfa Zhang

**Affiliations:** ^1^ Department of Ophthalmology of Tongji Hospital and Laboratory of Clinical and Visual Sciences of Tongji Eye Institute, Tongji University School of Medicine, Shanghai, China; ^2^ Department of Ophthalmology, Shanghai General Hospital (Shanghai First People’s Hospital), Shanghai Jiao Tong University School of Medicine, Shanghai, China; ^3^ National Clinical Research Center for Eye Diseases, Shanghai Key Laboratory of Ocular Fundus Diseases, Shanghai Engineering Center for Visual Science and Photomedicine, Shanghai Engineering Center for Precise Diagnosis and Treatment of Eye Diseases, Shanghai, China

**Keywords:** diabetic retinopathy, inner blood–retinal barrier, melatonin, microglia, pericyte, vascular endothelial cells

## Abstract

Microglial activation and melatonin protection have been reported in diabetic retinopathy (DR). Whether melatonin could regulate microglia to protect the inner blood–retinal barrier (iBRB) remains unknown. In this study, the role of microglia in iBRB breakdown and the mechanisms of melatonin’s regulation on microglia were explored. In diabetic rat retinas, activated microglia proliferated and migrated from the inner retina to the outer retina, accompanied by the obvious morphological changes. Meanwhile, significant leakage of albumin was evidenced at the site of close interaction between activated microglia and the damaged pericytes and endothelial cells. *In vitro*, inflammation-related cytokines, such as tumor necrosis factor-α (TNF-α), inducible nitric oxide synthase (iNOS), interleukin (IL)-1β, and arginase-1 (Arg-1), were increased significantly in CoCl_2_-treated BV2 cells. The supernatant derived from CoCl_2_-treated BV2 cells significantly decreased the cell viability and disrupted the junctional proteins in both pericytes and endothelial cells, resulting in severe leakage. Melatonin suppressed the microglial overactivation, i.e., decreasing the cell number and promoting its anti-inflammatory properties in diabetic rat retinas. Moreover, the leakage of iBRB was alleviated and the pericyte coverage was restored after melatonin treatment. *In vitro*, when treated with melatonin in CoCl_2_-treated BV2 cells, the inflammatory factors were decreased, while the anti-inflammatory factors were increased, further reducing the pericyte loss and increasing the tight junctions. Melatonin deactivated microglia *via* inhibition of PI3K/Akt/Stat3/NF-κB signaling pathways, thus maintaining the integrity of iBRB. The present data support a causal role for activated microglia in iBRB breakdown and highlight the therapeutic potential of melatonin in the treatment of DR by regulating microglia.

## Introduction

Diabetic retinopathy (DR) is the leading cause of the visual impairment in working-age people worldwide, which has been characterized as a neurovascular disease. In early DR, microglial activation has been shown to play a pivotal role resulting in the breakdown of the blood–retinal barrier (BRB) (1).

In the normal retinas, ramified microglia mainly distribute in the never fiber layer (NFL) and ganglion cell layer (GCL) (2), which are required for retinal development, vascular formation, retinal function, neurogenesis, and aging (3). Microglia also interact with other neurons, glial cells, and endothelial cells by producing growth factors and neuroprotective mediators (4), to maintain the homeostasis of retina. However, noxious insults such as inflammation, hypoxia, or oxidative stress trigger microglial activation, which is manifested by the amoeboid morphology, increased proliferation, and enhanced migration to the sites of injury (5, 6). Besides, the activated microglia secrete pro-inflammatory cytokines, such as tumor necrosis factor-α (TNF-α), interleukin (IL)-1β, IL-6, and chemotactic factors, as well as high levels of anti-inflammatory cytokines (7). The balance between pro-inflammatory and anti-inflammatory effects is hazy, and usually microglia can adapt their phenotypes to meet the demands (8).

Pericytes and endothelial cells, consisting of the retinal microvascular system, cooperatively form an intact inner BRB (iBRB) to maintain retinal homeostasis. However, pericyte loss and endothelial apoptosis, assumed as early events in DR, cause acellular capillaries and subsequent BRB breakdown (9). Meanwhile, activated microglia adapt their morphology from a ramified to an amoeboid form with enhanced migration to the outer plexiform layer (OPL) and photoreceptor layer (3, 10). Due to long-term activation of microglia, sustained production of inflammatory cytokines would instigate severe alterations in vascular dysfunction and aggravate neuronal death (1, 11).

Melatonin, a synthetic product of the pineal gland, is also produced locally in the retina to regulate dopamine metabolism and redox reactions (12, 13). Melatonin and its metabolites have been widely studied to exert both physiological functions and protection against inflammation and oxidative stress (14–16), such as suppressing the activity of pro-oxidant enzymes, including inducible nitric oxide synthase (iNOS), cyclooxygenase-2 (COX-2), and myeloperoxidase, as well as inhibiting multiple inflammatory factors, like TNF-α, intercellular adhesion molecule-1 (ICAM-1), and IL-1β. Considering its anti-inflammatory and antioxidative properties (17), it is reasonable to speculate that melatonin might exert beneficial effects on DR, especially by regulating microglial activity and maintaining the integrity of iBRB.

Currently, there are limited studies investigating the underlying mechanisms of melatonin’s regulation on microglia to protect iBRB in DR. In this study, we explored the possible mechanisms of melatonin to maintain iBRB by deactivating microglia in experimental DR both *in vivo* and *in vitro*.

## Methods

### Diabetic Rat Model and Intravitreal Melatonin Injection

All experiments adhered to the ARVO Statement for the Use of Animals in Ophthalmic and Vision Research, and the protocols were approved by the National Institutes of Health guide for the care and use of Laboratory animals (NIH Publications No. 8023) and the Committee on the Ethics of Animal Experiments of Tongji University (Permit Number: TJHBLAC-2021-06). About 75 male Sprague-Dawley rats weighing 120–150 g were purchased from the Shanghai Laboratory Animal Center (Chinese Academy of Sciences, Shanghai, China) and maintained on a 12:12-h light/dark cycle with free access to food and water. To establish diabetes, the rats were fasted for 24 h and intraperitoneally injected with streptozotocin (STZ, 60 mg/kg), while control animals were injected with the equivalent volume of phosphate-buffered saline (PBS). Information for blood glucose level and body weight of the rats is provided in **Supplementary Table S1**. Rats with blood glucose levels higher than 16.7 mmol/l for 3 consecutive days after injection were considered as diabetic. At 2 h after STZ injection, the right eye of rats was intravitreally injected with melatonin (30 μg/eye, 2 μl; Sigma-Aldrich), while the left eye was injected with the equivalent volume of normal saline. Intravitreal injection of melatonin was repeated every week based on our previous study (18), and the rats were killed at 8 weeks after diabetes onset. The rats that failed to develop diabetes and with intraocular damages (e.g., traumatic cataract, intraocular hemorrhage, inflammation) were excluded from the experiments.

### BV2 Cells, Human Retinal Microvascular Endothelial Cells, and Human Brain Vascular Pericyte Cultures

The BV2 cells, a mouse microglial cell line (American Type Culture Collection, Manassas, VA, USA), were cultured with high-glucose (25 mmol/l) DMEM containing 10% (vol./vol.) fetal bovine serum (FBS, Cat. #10091148; Gibco, NY, USA) and 1% (vol./vol.) penicillin/streptomycin (p/s, Cat. #15140122; Invitrogen, CA, USA). Human retinal microvascular endothelial cells (HRMECs; ScienCell Research Laboratories, Carlsbad, CA, USA) were cultured with endothelial culture medium (Cat. #1001; ScienCell) containing 5% (vol./vol.) FBS (Cat. #0025; ScienCell), 1% (vol./vol.) endothelial cell growth supplement (Cat. #1052; ScienCell), and 1% (vol./vol.) p/s solution (Cat. #0503; ScienCell). Human brain vascular pericytes (HBVPs; ScienCell Research Laboratories, Carlsbad, CA, USA) were cultured with pericyte culture medium (Cat. #1201; ScienCell) containing 2% (vol./vol.) FBS (Cat. #0010; ScienCell), 1% (vol./vol.) pericyte growth supplement (Cat. #1252; ScienCell), and 1% (vol./vol.) p/s solution (Cat. #0503; ScienCell). All cells were cultured at 37°C and 5% CO_2_ in a humidified incubator.

BV2 cells were divided into four groups, i.e., normal control, melatonin treatment, CoCl_2_ treatment, and CoCl_2_+melatonin treatment. CoCl_2_ prevents hypoxia inducible factor-1α (HIF-1α) from being hydroxylated by replacing the prolyl hydroxylase cofactor ferrous ion (19). The replacement of ferrous iron by CoCl_2_ affects the binding of von Hippel-Lindau protein to the oxygen-dependent degradation domain of HIF-1α, thus preventing HIF-1α degradation and stabilizing HIF-1α protein. In this study, BV2 cells were treated with CoCl_2_ to achieve similar effects as under hypoxia incubation.

To investigate the mechanism of melatonin on microglia, BV2 cells were pretreated with 50 μM luzindole (antagonist of melatonin receptor, Sigma-Aldrich), 100 nM MK2206 (Akt inhibitor, Selleck, Houston, TX, USA), or 20 μM SN50 (NF-κB inhibitor, Sigma-Aldrich, St. Louis, MO, USA) for 1 h before exposure to CoCl_2_ (100 μM, Sigma-Aldrich) with or without melatonin (100 μM) for 12 h.

**Figure 1:**
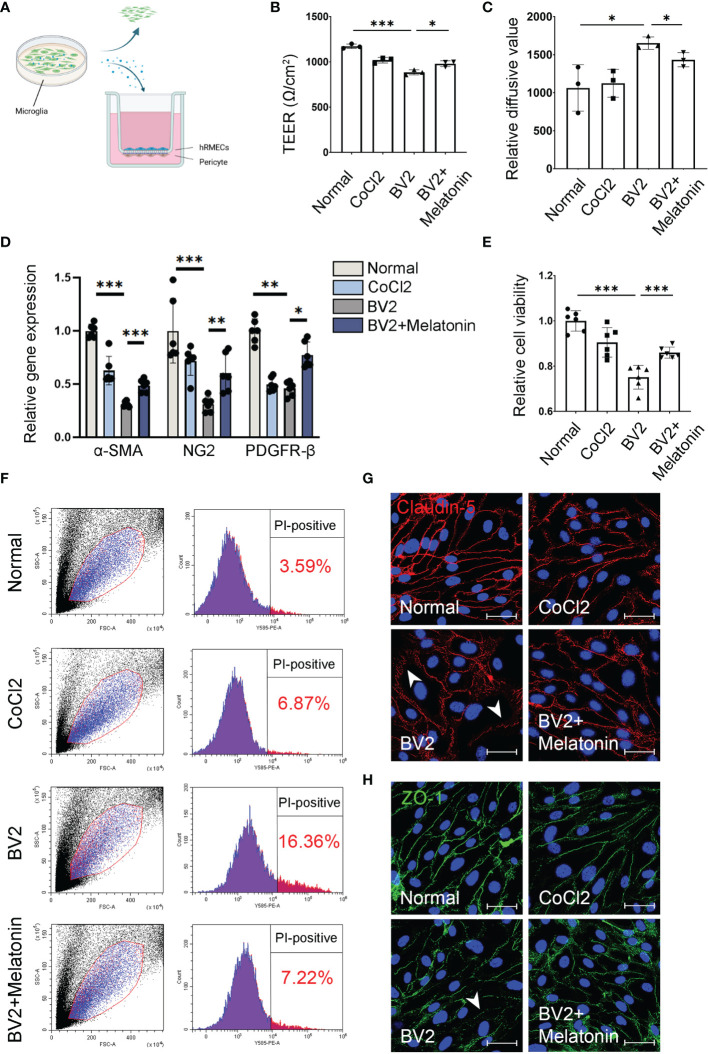
Melatonin protects the barrier function of pericytes and endothelial cells *in vitro.* **(A)** HBVPs were cocultured with HRMECs for 72 h, and then the supernatant of CoCl_2_-treated BV2 cells was added into the upper chamber of transwell. **(B)** The transendothelial electrical resistance (TEER) was detected in the HBVP/HRMEC coculture system under different conditions (n = 3 per group). **(C)** The FITC-dextran leakage was detected in the HBVP/HRMEC coculture system under different conditions (n = 3 per group). **(D)** The expressions of pericyte markers, including α-SMA, NG2, and PDGFR-β in CoCl_2_-treated HBVPs with or without melatonin for 12 h (n = 6 per group). **(E)** Cell viability of CoCl_2_-treated HBVPs with or without melatonin for 12 h (n = 6 per group). **(F)** Cell death of pericytes in CoCl_2_-treated HBVPs with or without melatonin for 12 h (n = 3 per group). **(G, H)** Representative images of Claudin-5 (red) and ZO-1 (green) immunostaining in CoCl_2_-treated HRMECs with or without melatonin. The nuclei were stained with DAPI (blue). Data are expressed as mean ± SEM. *p < 0.05, **p < 0.01, ***p < 0.001 by one-way ANOVA. Scale bar, 40 μm.

To study the causal effects of the inflammatory factors released by activated microglia on retinal capillaries in DR, we established an HBVP/HRMEC coculture system and then treated with the supernatant from CoCl_2_-treated microglia (**Figure 1A**). First, the basal side of the transwell membrane (Transwell; Corning Costar, Millipore, MA) was pre-coated with fibronectin to simulate the basement membrane of capillaries, and then HBVPs were seeded onto the basal side of the filter, while the HRMECs were seeded onto the apical side and cocultured for 72 h until they reached confluence. Then the supernatant of CoCl_2_-treated microglia with or without melatonin for 12 h was added into the apical side of the coculture system. The HBVP/HRMEC coculture directly exposed to CoCl_2_ for 12 h served as a positive control group.

### Immunofluorescence

The immunostaining method for retinal flatmounts was adopted from our previous study (1). Briefly, the retinal flatmounts were incubated with anti-IBA-1 (1:500, Cat. #019-19741; WAKO, Osaka, Japan), anti-NG2 (1:500, Cat. #37-2700; Invitrogen, Carlsbad, CA, USA), and anti-albumin (1:100, Cat. #66051; Proteintech, Wuhan, China) antibodies for 3 days. After three washes with 1× PBS for 10 min, the retinas were incubated with the corresponding secondary antibodies or fluorescent probe overnight in the dark. Then, the flatmounts were washed three times with 1× PBS and mounted with coverslips using Fluoromount-G (Cat. #0100-01; SouthernBiotech, AL, USA) followed by examination under a confocal microscope (Leica DMI3000). For the immunofluorescence on retinal cryosections and cell samples, the cryosections or cells were permeabilized with blocking solution consisting of 1% (wt/vol.) BSA and 0.05% (vol./vol.) Triton X-100 in 1× PBS for 1 h at room temperature and then incubated with the corresponding antibodies, e.g., anti-IBA-1, anti-MT-1, anti-CD16/32, anti-CD206, anti-ZO-1, or anti-Claudin-5 antibodies, overnight at 4°C. After washing three times with 1× PBS for 10 min, the sections were incubated with the corresponding secondary antibodies for 1 h at room temperature. After three washes with 1× PBS, the sections were mounted with DAPI-Fluoromount-G (Cat. #0100-20; SouthernBiotech) and examined under a confocal microscope. The antibody information is listed in **Table S2**.

### Western Blot

Cells and retinas were lysed with radioimmunoprecipitation assay lysis buffer (Cat. #20101ES60; Yeasen, Shanghai, China) containing a protease inhibitor cocktail (1:50; Roche, Basel, Switzerland) and phosphatase inhibitor (1:10; Roche). The samples were sonicated for 10 s and then placed on ice for 30 min. The protein concentration was measured with the Pierce Bicinchoninic Acid Protein Assay Kit (Cat. #23225; Thermo Fisher Scientific, MA, USA). After denaturation at 100°C for 5 min, proteins were separated by SDS-PAGE and probed with the corresponding primary and secondary antibodies (**Table S2**). Protein bands were detected by chemiluminescence with the imaging system (Tanon 5200, Shanghai, China).

### Transendothelial Electrical Resistance Measurement

Transendothelial electrical resistance (TEER) was performed in the HBVP/HRMEC coculture system with the ERS-2 (Electrical Resistance System, Millicell) according to the manufacturer’s instructions. After reaching confluence, the HBVP/HRMEC coculture was treated with the supernatant from CoCl_2_-treated microglia with or without melatonin treatment for 12 h. Measurements were performed four times in quadruplicate within the HBVP/HRMEC coculture system, and TEER was presented as absolute values (Ohm × cm^2^).

### Cell Permeability Assay

The FITC-dextran was performed in the HBVP/HRMEC coculture system. After reaching confluence, the cells were treated with the supernatant from CoCl_2_-treated microglia with or without melatonin treatment for 12 h, and then FITC-dextran (70 kDa, 100 μg/ml, Sigma-Aldrich) was added into the upper chamber of the transwell. After 1 h, 200 μl medium was collected from the lower chamber and the absorbance was measured at an excitation wavelength of 485 nm and an emission wavelength of 528 nm with a multifunctional microplate reader (BioTek Synergy 4, Winooski, VT, USA). Concentrations were calculated from the standard curve.

### Cell Viability Assay

Cell viability was measured with the Cell Counting Kit-8 (CCK-8) assay. HBVPs were seeded on 96-well plates at a density of 1.0 × 10^5^ cells per well and cultured overnight. After reaching confluence, the cells were treated with the supernatant from CoCl_2_-treated microglia with or without melatonin for 12 h. The HBVPs, directly exposed to CoCl_2_ for 12 h, served as a positive control group. Then the medium was replaced with fresh medium containing 0.5 mg/ml CCK-8 for 4 h. The absorbance was measured at 450 nm with a microplate spectrophotometer (Tecan, Crailsheim, Germany).

### Quantitative Reverse Transcription Polymerase Chain Reaction

Total RNA was extracted, and reverse transcription was performed using the PrimeScript RT Master Mix Kit (Takara, Shiga, Japan). Real-time PCR was performed using the SuperReal PreMix Plus (SYBR Green) Kit (Tiangen Biotech, Beijing, China). The relative expression levels of the different genes were calculated using the 2^−ΔΔCt^ method with β-actin as the internal control. The primer sequences are shown in **Table S3**.

### Flow Cytometry

Pericytes were co-stained with Annexin V and propidium iodide (Cat. #40305ES20; Yeasen). After three washes, the cells were assayed by flow cytometry, and the ratio of apoptotic or necrotic cells to normal control was calculated accordingly.

### Bioinformatics Analysis

Microglial gene profiles (GSE148350) downloaded from Gene Expression Omnibus (GEO) were analyzed to explore the differentially expressed genes. The heatmap and the volcano plot of statistical genes were constructed with cutoff criteria of log_2_FC > 1.0 and adj. p value < 0.05. Gene Ontology (GO) and Kyoto Encyclopedia of Genes and Genomes (KEGG) pathways were analyzed by David online database (https://david.ncifcrf.gov/). Metascape utilizes the well-adopted hypergeometric test and Benjamini–Hochberg p-value correction algorithm to identify all ontology terms that contain a greater number of genes in common with an input list than expected by chance. The STRING online database (https://string-db.org/) was applied to construct a protein–protein interaction (PPI) network of microglial activation genes.

### Statistical Analysis

All data were expressed as mean ± SEM and analyzed with the one-way ANOVA and SNK-q using IBM SPSS Statistics (Version 26; IBM, USA) and GraphPad Prism 8 (GraphPad Software, La Jolla, CA, USA). The differences for body weight or blood glucose in **Table S1** were analyzed with two-tailed Student’s t test (unpaired t test). A *p* value of 0.05 or less was considered statistically significant.

## Results

### Melatonin Inhibits Microglial Activation in the Experimental DR

**Figure 2:**
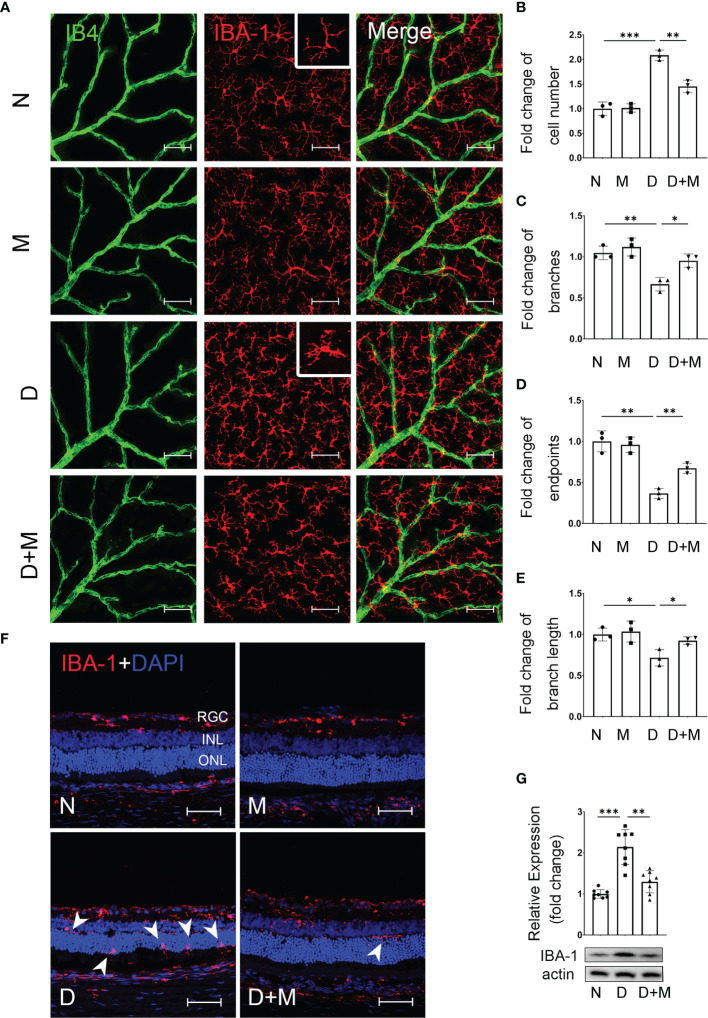
Melatonin inhibits microglial activation in diabetic rat retinas. **(A)** Representative images of IBA-1 (red) and IB4 (green) immunolabeling in superficial capillary plexus of the retinal flatmounts in normal control and diabetic rat retinas with or without melatonin treatment. **(B–E)** Comparison of morphological changes in microglial number, cell branches, cell endpoints, and cell branch length in normal control and diabetic rat retinas with or without melatonin treatment. **(F)** Representative images of IBA-1 (red) and DAPI (blue) immunolabeling of retinal cryosections in normal control and diabetic rat retinas with or without melatonin treatment. Microglial migration is indicated by white arrowheads (n = 3 retinas per group). **(G)** IBA-1 protein expression in normal control and diabetic rat retinas with or without melatonin treatment. Data are expressed as mean ± SEM (n = 8 retinas per group). N, normal control group; M, melatonin treatment group; D, diabetes group; D+M, diabetes with melatonin treatment group; *p < 0.05, **p < 0.01, ***p < 0.001 by one-way ANOVA. Scale bar, 50 μm.

To detect the changes of microglia and their relationship with retinal capillaries in the experimental DR, we performed co-immunostaining of IBA-1 (microglial marker) and isolectin B4 (IB4, endothelial cell marker) on retinal flatmounts. In normal control, the microglia, displaying the ramified morphology, were distributed evenly in retina, while they became activated in the diabetic group, showing the characteristic amoeboid morphology and close relationship with retinal capillaries (**Figure 2A**). To further characterize microglial activation, we analyzed the changes in cell number, branch points, endpoints, and branch length of microglia in the diabetic rat retinas treated with or without melatonin. As shown in **Figures 2B**–**E**, in diabetic rat retinas, the number of microglia in superficial capillary plexus was about 2.08-fold of the normal control (n = 3, p < 0.001), whereas the branch points, endpoints, and branch length of microglia were decreased significantly by 36.36% (n = 3, p = 0.004), 63.27% (n = 3, p = 0.001), and 29.43% (n = 3, p = 0.01), respectively, compared with the normal control. The migration of microglia was also enhanced in the diabetic group (**Figure 2F**). In normal control, the microglia were mainly distributed in the inner retina, e.g., from the nerve fiber layer (NFL) to the inner plexiform layer (IPL), while they migrated to the outer retina, e.g., the outer plexiform layer (OPL), and even the subretinal space and retinal pigment epithelial layer, in the diabetic group. Besides histological analysis, to further confirm microglial activation in the diabetic retina, the protein expression of IBA-1 was detected with Western blot and demonstrated that, compared with the normal control, the IBA-1 protein level in the diabetic retinas was significantly increased, about 2.02-fold that of the normal control (p < 0.001, **Figure 2G**).

When treated with melatonin, the microglia were deactivated as shown in **Figures 2A**–**G**. Compared with that in the diabetic group, melatonin treatment decreased the cell number by 30.41% (n = 3, p = 0.002), while it increased the number of branch points, endpoints, and branch length by 42.86% (n = 3, p = 0.013), 83.33% (n = 3, p = 0.003), and 29.02% (n = 3, p = 0.03), respectively (**Figures 2A**–**E**). Moreover, the migration of microglia was reduced (**Figure 2F**) and the IBA-1 protein expression was also decreased by 35.99% (n = 8, p = 0.001) by melatonin (**Figure 2G**).

### Melatonin Alleviates Diabetes-Induced Retinal Vascular Leakage and Endothelial Cell Damage

**Figure 3:**
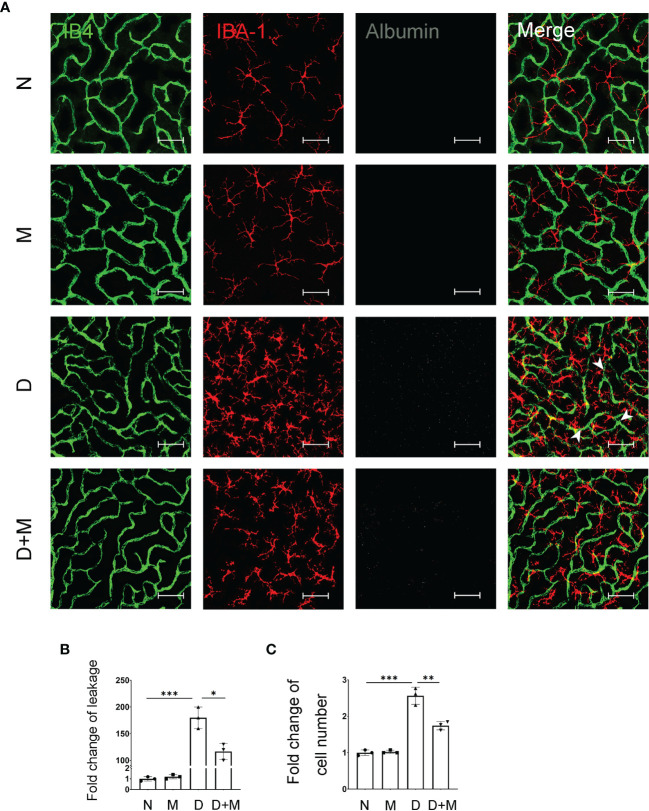
Melatonin alleviates diabetes-induced retinal vascular leakage and endothelial cell damage. **(A)** Representative images of IB4 (green), IBA-1 (red), and albumin (white) immunolabeling in deep capillary plexus of the retinal flatmounts in normal control and diabetic rat retinas with or without melatonin treatment. Leaked albumin is indicated by white arrowheads. **(B)** Quantification of albumin leakage in the retinas. **(C)** Quantification of microglial number in normal control and diabetic rat retinas with or without melatonin treatment. Data are expressed as mean ± SEM (n = 3 retinas per group). N, normal control group; M, melatonin treatment group; D, diabetes group; D+M, diabetes with melatonin treatment group; *p < 0.05, **p < 0.01, ***p < 0.001 by one-way ANOVA. Scale bar, 50 μm.

In our previous study, we reported that activated microglia phagocytosed retinal endothelial cells, resulting in the breakdown of iBRB in experimental DR (1). Here, we confirmed the activation of microglia and breakdown of iBRB with the co-immunostaining of IB4, IBA-1, and albumin in the retinal deep capillary plexus. Compared with normal control, the albumin leaked significantly into the retinal parenchyma in the diabetic retinas, indicating the breakdown of iBRB (**Figures 3A, B**). In addition, the microglia became activated showing the increased number of amoeboid microglia. As in deep capillary plexus, the activated microglia in diabetic rat retinas were about 2.52-fold (n = 3, p < 0.001) of normal control, which mainly gather around the retinal capillaries (**Figures 3A, C**). Surprisingly, melatonin treatment alleviated the albumin leakage by 35.19% (p = 0.01) and decreased the microglial number by 32.00% (p = 0.005).

### Melatonin Prevents Retinal Pericyte Loss in the Experimental DR

**Figure 4:**
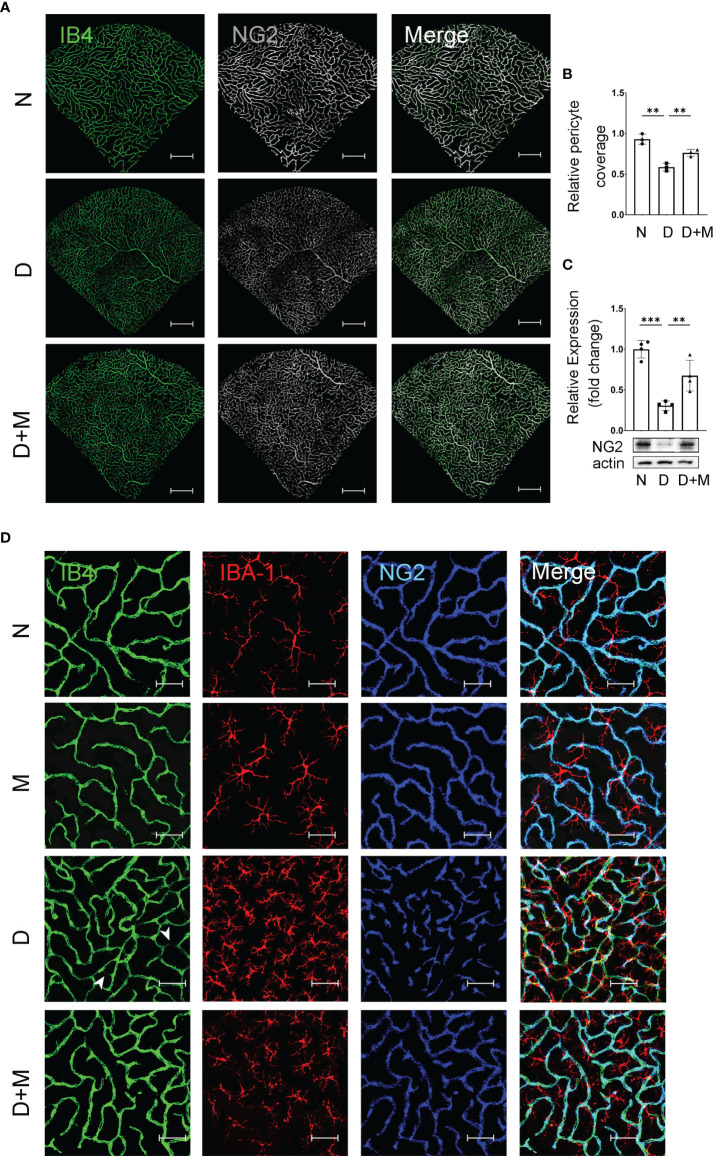
Melatonin prevents retinal pericyte loss in diabetes retinopathy. **(A)** Representative images of IB4 (green) and NG2 (gray) immunolabeling in normal control and diabetic rat retinas with or without melatonin treatment. **(B)** Quantification of pericyte coverage. **(C)** NG2 expression in normal control and diabetic rat retinas with or without melatonin treatment (n = 4 retinas per group). **(D)** Representative images of IB4 (green), IBA-1 (red), and NG2 (blue) immunolabeling in normal control and 8-week diabetic rat retinas with or without melatonin treatment. Endothelial loss is indicated by white arrowheads. Data are expressed as mean ± SEM (n = 3 retinas per group). N, normal control group; M, melatonin treatment group; D, diabetes group; D+M, diabetes with melatonin treatment group; **p < 0.01, ***p < 0.001 by one-way ANOVA. Scale bar, 50 μm.

Pericyte loss is an early pathological feature of DR. To detect if melatonin treatment could prevent pericyte loss in DR, we performed immunostaining and Western blot. The co-immunostaining of IB4 and NG2 (pericyte marker) showed that the pericyte coverage in diabetic retinas was decreased significantly by 36.99% (n = 3, p = 0.002) compared with the normal group (**Figures 4A, B**). The pericyte loss was further confirmed with Western blot; the NG2 protein level in the diabetic group decreased by 69.37% (n = 4, p < 0.001) compared with the normal group (**Figure 4C**). We further studied whether microglial activation was related to diabetes-induced retinal vascular damage. As shown in **Figure 4D**, compared with the normal group, diabetic rat retinas showed incomplete pericyte coverage and endothelial cell degeneration accompanied by the activated microglia, especially gathering the site of injured vessels. Besides deactivating the microglia, melatonin treatment could also increase the coverage of pericyte by 29.93% (n = 3, p = 0.008) and the NG2 protein level by 1.2-fold (n = 4, p = 0.009), compared with the diabetic group (**Figure 4**).

### Melatonin Receptor MT1 Was Expressed in Microglia

**Figure 5:**
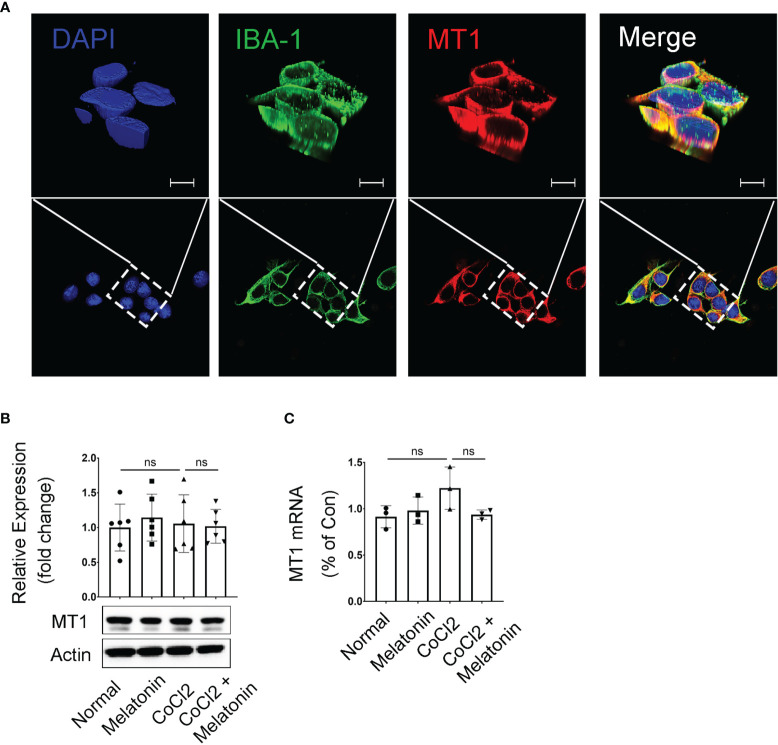
Melatonin receptor expression in CoCl_2_-treated BV2 cells. **(A)** Representative images of MT1 (red) and IBA-1 (green) immunostaining in microglia. Nuclei were stained with DAPI (blue). **(B, C)** MT1 proteins (n = 6 per group) and mRNA (n = 3 per group) expression in CoCl_2_-treated BV2 cells with or without melatonin treatment. Data are expressed as mean ± SEM. ns, non-significant difference. Scale bar, 10 μm.

The above data demonstrated the microglial activation and the pathological changes of retinal capillaries in the experimental DR, as well as the protective effects of melatonin to reverse the above conditions. Thus, it is reasonable to hypothesize that melatonin might prevent the breakdown of iBRB by deactivating the microglia in diabetic retina. To prove this, we first investigated the expression and localization of the melatonin receptor (MT1) in BV2 cells, a mouse microglial cell line. The immunostaining showed that MT1 was mostly located on the membrane of microglia, partially colocalized with the microglial marker IBA-1 (**Figure 5A**). The mRNA and protein levels of MT1 remained relatively unchanged in both normal control and CoCl_2_-treated BV2 cells with or without melatonin treatment (**Figures 5B, C**). The existence of MT1 in microglia laid down the foundation for the regulation of melatonin on microglia.

### Melatonin Protects the Barrier Function of Endothelial Cells and Pericytes *In Vitro*

Beside direct phagocytosis of endothelial cells by activated microglia (1), the indirect effect between activated microglia and breakdown of iBRB, as well as the regulation of melatonin on iBRB, deserved exploration. To determine whether the activated microglia-derived inflammatory factors result in the breakdown of iBRB, we employed the coculture system of pericytes and endothelial cells and treated this system with the supernatant from CoCl_2_-treated BV2 cells (**Figure 1A**). TEER and FITC-dextran assay showed that the barrier function of pericytes and endothelial cells was damaged when treated with the supernatant from CoCl_2_-treated BV2 cells. Compared with the normal control, TEER was decreased by 24.55% (n = 3, p < 0.001, **Figure 1B**), and the FITC-dextran leakage was increased by 55.45% (n = 3, p = 0.03, **Figure 1C**) in the group of the supernatant from CoCl_2_-treated BV2 cells. When treated with melatonin, the damaging effect on the barrier function by the supernatant from CoCl_2_-treated BV2 cells was largely compromised, i.e., increased by 10.80% (TEER, n = 3, p = 0.018, **Figure 1B**) and decreased by 13.22% (FITC-dextran leakage, n = 3, p = 0.03, **Figure 1C**).

As for the indirect effect of activated microglia on pericytes, the expressions of pericyte markers, including α-smooth muscle actin (α-SMA), NG2, and platelet-derived growth factor receptor-β (PDGFR-β), were detected in pericyte culture treated with CoCl_2_-treated BV2 supernatant with or without melatonin incubation. As shown in **Figure 1D**, the mRNA expressions of α-SMA, NG2, and PDGFR-β in HBVPs were decreased by 69.04% (n = 6, p < 0.001), 69.11% (n = 6, p < 0.001), and 54.35% (n = 6, p = 0.008), respectively, under the treatment of the supernatant from CoCl_2_-treated BV2 cells, while melatonin pre-incubation increased the mRNA expressions of α-SMA, NG2, and PDGFR-β of HBVPs by 57.46% (n = 6, p < 0.001), 95.49% (n = 6, p = 0.003), and 69.61% (n = 6, p = 0.02), respectively. To further confirm the changes of pericytes under different conditions, the cell viability and cell death of pericytes were analyzed with CCK-8 assays and propidium iodide (PI) staining. The data revealed that CoCl_2_-treated BV2 supernatant decreased significantly the viability of HBVPs by 24.90% (n = 6, p < 0.001) and increased cell death by 4.56-fold (PI-positive cells) compared with normal control (**Figures 1E, F**). Melatonin treatment reversed the above conditions, i.e., increased cell viability by 14.48% (n = 6, p = 0.001) and decreased cell death by 55.87% (n = 3, p < 0.05), demonstrating that melatonin increased cell viability and decreased cell death by directly deactivating microglia.

Besides HBVPs, to further confirm the maintenance of the barrier function exerted by the regulation of melatonin on microglia, the immunostaining of tight junctions was performed on HRMECs under similar conditions. Consistent with these observations, the immunostainings of ZO-1 and Claudin-5 in HRMECs treated with the supernatant derived from CoCl_2_-treated BV2 cells were largely disrupted compared with the normal control, which were restored after melatonin treatment (**Figures 1G, H**).

### Microglial Transcriptome Analysis Reveals PI3K/Akt/Stat3/NF-κB Pathways in an Ischemic Rat Model

**Figure 6:**
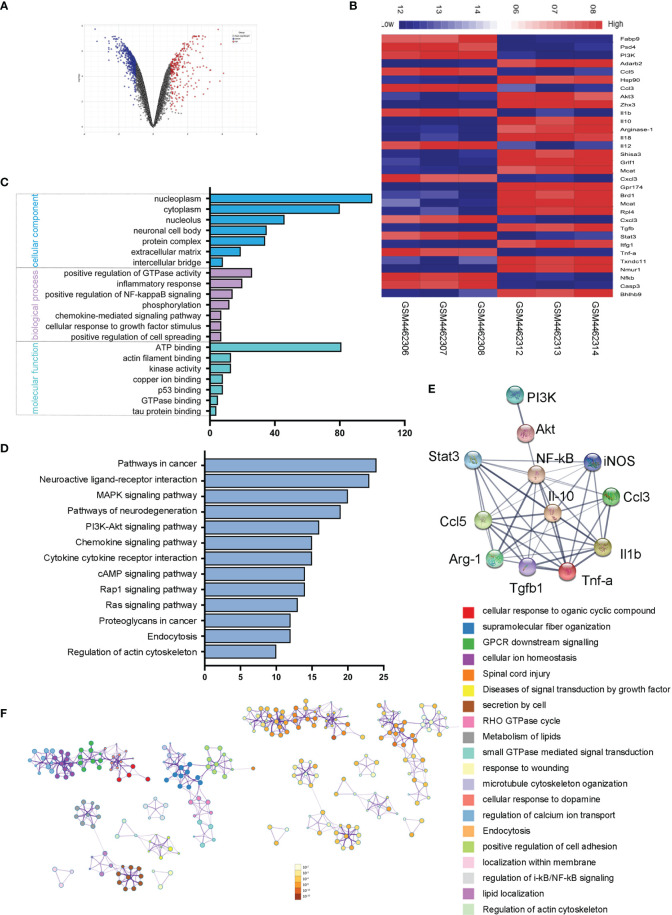
Microglial transcriptome analysis in an ischemic rat model. **(A)** Volcano plot of differentially expressed genes in the stroke rat samples compared with those of normal samples from the GEO dataset. Red and blue indicate upregulated and downregulated, respectively. **(B)** The heatmap shows 32 genes from top 50 differentially expressed genes of microglial activation. Red and blue represent the upregulated and downregulated genes. **(C)** Differentially expressed genes of microglial activation are enriched by GO analysis including biological process, molecular function, and cellular component. **(D)** KEGG pathways enrichment for upregulated genes. **(E)** The PPI network of microglia-activated genes constructed by STRING. **(F)** Functional annotation of significantly expressed genes using Metascape. Orange denotes the enrichment terms colored by p-values and an interactive network of the enrichment terms colored by cluster ID.

To explore the sequential molecular and cellular events triggered by microglial activation, we analyzed and compared the microglial gene expressions in ischemic rats with those of normal controls. We screened out 272 upregulated genes and 585 downregulated genes with the fold change > 1 and adjusted p < 0.05. The volcano plot and heatmap of the differentially expressed genes were shown in **Figures 6A, B**. To further explore the potential changes of activated microglia, the GO terms for biological process (BP), cellular component (CC), and molecular function (MF), as well as KEGG pathways, were analyzed to reveal the biological functions of the differential expressed genes of microglia. The results showed that the GO terms were primarily enriched in NF-κB-mediated regulation, inflammatory response, cellular response to growth factor stimulus, and kinase activity (**Figure 6C**), and KEGG indicated that the microglia-related genes were mainly enriched in the PI3K-Akt signaling pathway, chemokine signaling pathway, and cytokine–cytokine receptor interaction (**Figure 6D**). A PPI network of microglia-related genes was generated using the STRING database (**Figure 6E**), and there were 53 edges and 12 nodes, including PI3K, Akt, Stat3, NF-κB, CCL3, and CCL-5. To visualize the significant pathways, Metascape’s functional enrichment analysis was further exploited. Each node represented an enriched term and was colored by the cluster ID. The enriched terms mainly included GPCR downstream signaling, diseases of signal transduction by growth factor, secretion by cell, small GTPase-mediated signal transduction, positive regulation of cell adhesion, and regulation of IκB kinase/NF-κB signaling (**Figure 6F**). Thus, the transcriptome analysis of the microglial activation in ischemic rats led to the PI3K/Akt/Stat3/NF-κB-related signaling pathways in the following study.

### Melatonin Deactivates Microglia by Balancing Pro‐Inflammatory and Anti‐Inflammatory Mediators

**Figure 7:**
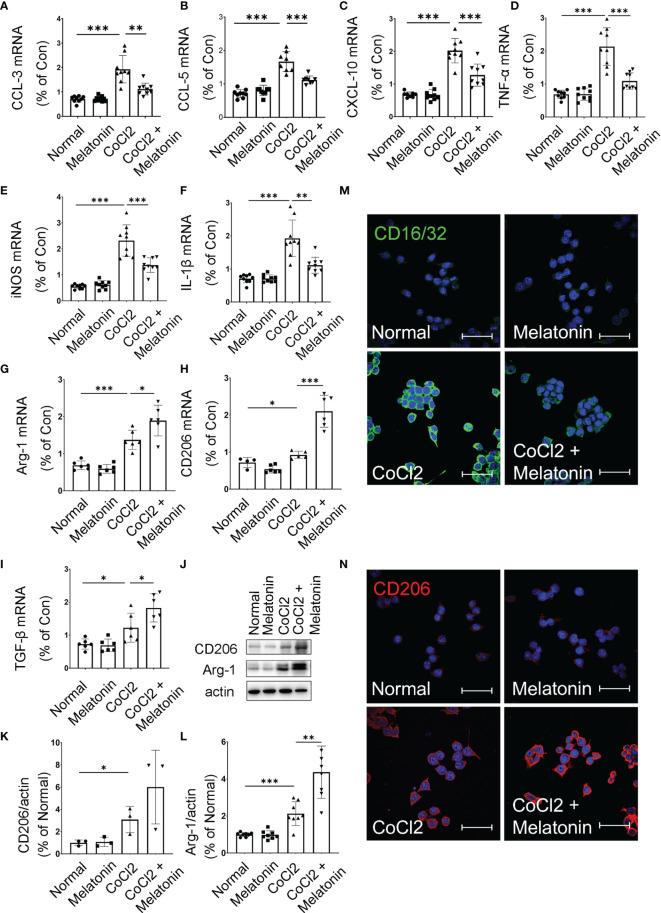
Melatonin regulates microglial activity by balancing pro-inflammatory and anti-inflammatory mediators. The mRNA expressions of CCL-3 **(A)**, CCL-5 **(B)**, CXCL-10 **(C)**, TNF-α **(D)**, iNOS **(E)**, IL-1β **(F),** Arg-1 **(G)**, CD206 **(H)**, and TGF-β **(I)** in the control group, control + melatonin group, CoCl_2_ group, and CoCl_2_ + melatonin group (n = 6 per group). **(J–L)** The protein expressions of CD206 and Arg-1 in the control group, control + melatonin group, CoCl_2_ group, and CoCl_2_ + melatonin group (n = 3 per group). **(M, N)** Representative images of CD16/32 (green) and CD206 (red) immunostaining in CoCl_2_-treated microglia with or without melatonin. The nuclei were stained with DAPI (blue). Data are expressed as mean ± SEM; *p < 0.05, **p < 0.01, ***p < 0.001 by one-way ANOVA. Scale bar, 40 μm.

Considering bioinformatics analyses of microglial transcriptome in the ischemic rat models, we next investigated the inflammatory cytokines produced by the microglia under diabetic conditions. The mRNA expressions of pro-inflammatory and anti-inflammatory cytokines were detected in CoCl_2_-treated BV2 cells with or without melatonin treatment (**Figures 7A**–**I**). Similar to bioinformatic results, mRNA levels of both pro-inflammatory (CCL-3, CCL-5, CXCL-10, TNF-α, iNOS, and IL-1β) and anti-inflammatory (Arg-1, CD206, and TGF-β) markers were increased in CoCl_2_-treated BV2 cells compared with that in normal control, whereas melatonin treatment decreased pro-inflammatory cytokines and further increased anti-inflammatory cytokines, as compared to those in the CoCl_2_-treated group. Consistent with the mRNA results, the protein expressions of CD206 and Arg-1 increased in CoCl_2_-treated BV2 cells and further upregulated after melatonin treatment (**Figures 7J**–**L**). Besides, the microglial immunoreactivity for CD16/32 and CD206 was much stronger in the CoCl_2_-treated group than normal control, while melatonin treatment reduced CD16/32 and further enhanced CD206 (**Figures 7M, N**). The observations suggested that hypoxia induced by CoCl_2_ facilitated microglial activation, which increased the production of pro-inflammatory cytokines, possibly mediating the breakdown of iBRB, and melatonin deactivated microglia by reducing its pro-inflammatory production and enhancing the release of anti-inflammatory cytokines.

### Melatonin Inhibits PI3K/Akt/Stat3/NF-κB Signaling Pathways to Deactivate Microglia in the Experimental DR

**Figure 8:**
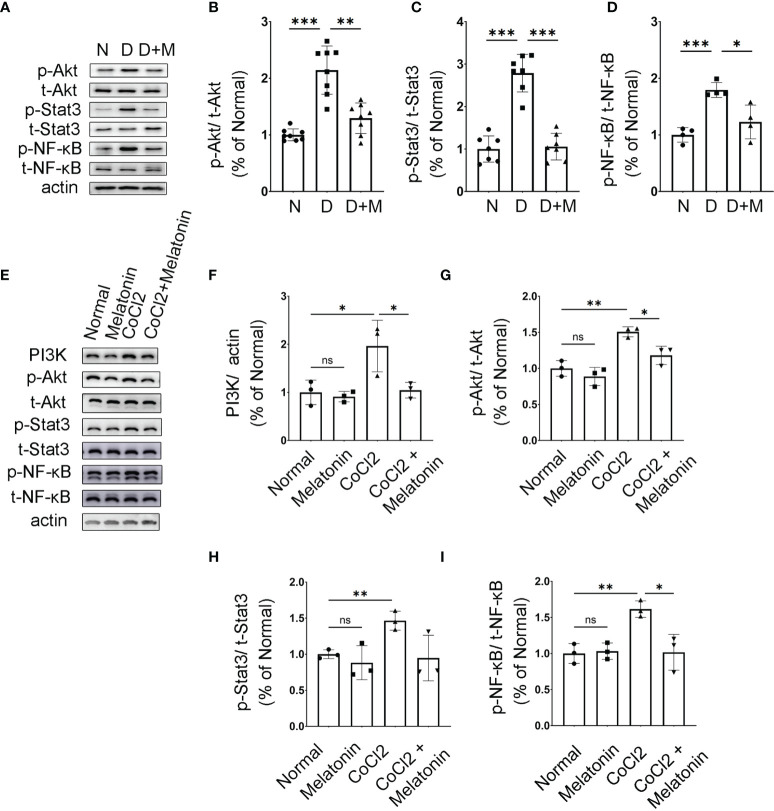
Melatonin inhibits AKT and Stat3 signaling pathways in activated microglia. **(A–D)** The protein expressions of p-Akt, p-Stat3, and p-NF-κB in normal control and diabetic rat retinas with or without melatonin treatment. Data are expressed as mean ± SEM (n = 4 retinas per group). **(E–I)** The protein expressions of PI3K, p-Akt, p-Stat3, and p-NF-κB in the control group, control + melatonin group, CoCl_2_ group, and CoCl_2_ + melatonin group. Data are expressed as mean ± SEM (n = 3 retinas per group); *p < 0.05, **p < 0.01, ***p < 0.001 by one-way ANOVA. ns, non-significant difference.

**Figure 9:**
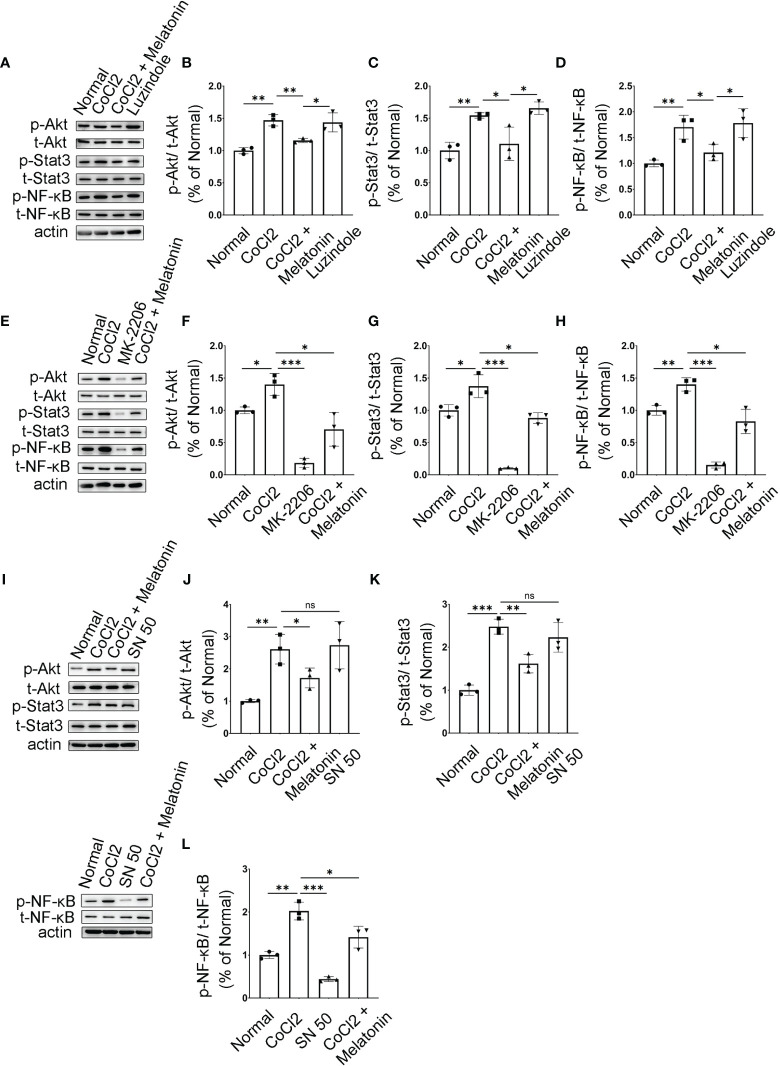
Melatonin inhibits PI3K/Akt/Stat3/NF-κB signaling pathways in CoCl_2_-treated microglia. **(A–D)** Protein expressions of p-Akt, p-Stat3, and p-NF-κB in the control group, CoCl_2_ group, CoCl_2_ + melatonin group, and luzindole. **(E–H)** Protein expressions of p-Akt, p-Stat3, and p-NF-κB in the control group, CoCl_2_ group, CoCl_2_ + melatonin group, and MK2206. **(I–L)** Protein expressions of p-Akt, p-Stat3, and p-NF-κB in the control group, CoCl_2_ group, CoCl_2_ + melatonin group, and SN50. Data are expressed as mean ± SEM (n = 3 per group); *p < 0.05, **p < 0.01, ***p < 0.001 by one-way ANOVA. ns, non-significant difference.

We then attempted to determine the molecular mechanisms and signaling pathways of microglial activation and melatonin regulation. Based on PPI analysis in the ischemic rat models, the protein expressions of PI3K, Akt, Stat3, and NF-κB were analyzed in diabetic rat retinas treated with or without melatonin. As shown in **Figures 8A**–**C**, the expressions of phosphor-Akt (p-Akt) and phosphor-Stat3 (p-Stat3) in diabetic rat retinas were all upregulated significantly, which were about 2.13- (p-Akt, n = 5, p < 0.001) and 2.78-fold (p-Stat3, n = 7, p < 0.001) that in the control group, and melatonin treatment decreased the protein levels by 29.97% (p-Akt, n = 5, p = 0.008) and 62.08% (p-Stat3, n = 7, p < 0.001). As NF-κB is one of the major components of hypoxia-induced signaling pathways and is involved in the production of pro-inflammatory factors and chemokines, the change of NF-κB was also detected in diabetic rat retinas with or without melatonin treatment. Similar to Akt and Stat3, p-NF-κB was upregulated significantly in diabetic rat retinas, i.e., 1.79-fold of the control group (**Figures 8A, D**), which was decreased by melatonin. The changes of p-Akt, p-Stat3, and p-NF-κB in diabetic rat retinas were validated *in vitro*. When microglia were treated with CoCl_2_, the protein expressions of PI3K, p-Akt, p-Stat3, and p-NF-κB were about 1.96-fold (n = 3, p = 0.048), 1.51-fold (n = 3, p = 0.002), 1.47-fold (n = 3, p = 0.005), and 1.62-fold (n = 3, p = 0.003), respectively, of that in the normal control (**Figures 8E**–**I**). The above changes were prevented by melatonin, i.e., decreased by 46.79% (PI3K, n = 3, p = 0.046), 21.71% (p-Akt, n = 3, p = 0.017), 35.35% (p-Stat3, n = 3, p = 0.058), and 37.06% (p-NF-κB, n = 3, p = 0.018), respectively (**Figures 8E**–**I**). Melatonin’s effect on microglial deactivation was largely abolished by luzindole, a non-specific melatonin receptor antagonist, indicating that melatonin exerted its regulation on microglia *via* its receptor (**Figures 9A**–**D**).

Microglia were pretreated with an Akt inhibitor, MK2206, for 1 h, and then stimulated with 100 μM CoCl_2_ for 12 h. MK2206 significantly reduced CoCl_2_-induced p-Akt, p-Stat3, and p-NF-κB upregulation (**Figures 9E**–**H**), showing that the PI3K/Akt signaling pathway is an upstream regulator of Stat3 and NF-κB. To see if NF-κB was the downstream target of Stat3, the NF-κB inhibitor (SN50) was adopted and examined in the same culture system. The results revealed that SN50 treatment had no effect on Akt and Stat3 (**Figures 9I**–**K**), while SN50 significantly inhibited CoCl_2_-induced phosphorylation of NF-κB (**Figures 9I, L**), indicating that NF-κB was the downstream target of both Akt and Stat3.

**Figure 10:**
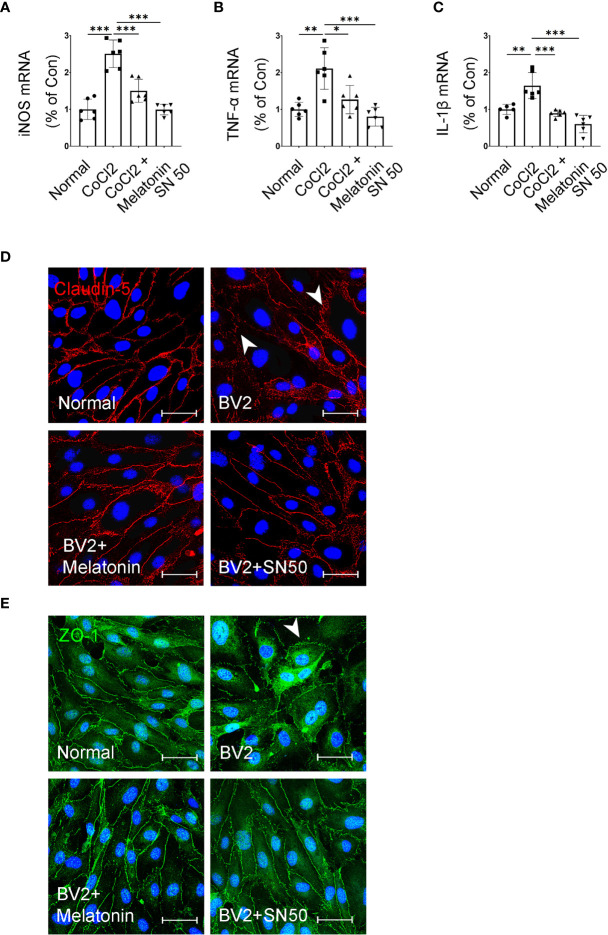
Melatonin suppresses the inflammatory factors in CoCl_2_-treated microglia and maintains the tight-junction proteins in endothelial cells. **(A–C)** The mRNA expressions of iNOS, TNF-α, and IL-1β in the control group, CoCl_2_ group, CoCl_2_ + melatonin group, and SN50. **(D, E)** Representative images of Claudin-5 (red) and ZO-1 (green) immunostaining in CoCl_2_-treated HRMECs with or without melatonin or SN50. The nuclei were stained with DAPI (blue). Data are expressed as mean ± SEM (n = 6 per group); *p < 0.05, **p < 0.01, ***p < 0.001 by one-way ANOVA.

In addition, SN50 was used to further substantiate the involvement of inflammation in iBRB disruption. As shown in **Figures 10A**–**C**, SN50 treatment inhibited CoCl_2_-induced mRNA expressions of TNF-α, iNOS, and IL-1β in BV2 cells, which was similar to the effect of melatonin. Meanwhile, the tight junctions between endothelial cells were also restored by the NF-κB inhibitor (**Figures 10D, E**). In comparison to the normal control, the distribution of ZO-1 and Claudin-5 was disrupted in HRMECs cells by the supernatant from the activated microglia, which was largely prevented by both melatonin and NF-κB inhibitor.

The present data suggested that melatonin inhibited the microglial production of inflammatory factors through its inhibition of NF-κB *via* suppressing PI3K/Akt/Stat3 signaling pathways, thus protecting the integrity of iBRB.

## Discussion

Recently, the role of microglia in accelerating vascular lesions has been widely investigated in DR. Activated microglia, manifested by enhanced phagocytic and migratory capacities, as well as cytokine release, regulate the microenvironment in diabetic retina (4, 7, 20). However, overactivated microglia could also interfere with retinal homeostasis and cause neuronal or vascular damages under constant inflammation and oxidative stress. A large body of evidence showed that excessive cytokine secretion and enhanced phagocytosis of the activated microglia have been involved in the pathogenesis of DR (11, 21–23), age-related macular degeneration (AMD), and retinal pigmentosa (3, 24). Moreover, coculture of microglia with endothelial cells under hypoxia resulted in endothelial cell phagocytosis *in vitro* (1). Therefore, these findings suggest the double-edged sword of microglial activation in DR.

The breakdown of iBRB, an early event of DR, is characterized by the apoptosis of endothelial cells and pericytes, progressive thickening of the basement membrane and acellular capillaries (25). Chronic inflammation exacerbates the iBRB breakdown since many inflammatory factors are upregulated in retinas and vitreous in DR patients, including IL-6, IL-1β, and TNF-α (26). Meanwhile, neuronal death and decreased neurotrophin, such as pigment epithelium-derived factor (PEDF), also induce vascular leakage (27–29). However, there are limited studies investigating the role of microglia in causing iBRB breakdown (30), and the regulation of melatonin on microglia and the possible mechanisms. In this study, we observed that the activated microglia proliferated and migrated from the inner to outer retina in diabetic rat retinas, accompanied by the morphological changes from ramified to amoeboid morphologies. Meanwhile, more acellular capillaries presented obvious leakage at sites in close touch with amoeboid microglia under diabetic conditions. Besides the direct effect on iBRB breakdown *via* phagocytosis (1), the inflammatory factors released by the activated microglia were also involved in iBRB breakdown in our study. In the HBVP/HRMEC coculture system, supernatant derived from the activated microglia injured both pericytes and endothelial cells, disrupted the tight-junctional proteins (Claudin-5 and ZO-1) between endothelial cells, and increased barrier leakage, indicating the indirect effect of activated microglia on iBRB breakdown (**Figure 1**).

Melatonin was demonstrated to contribute beneficial effects in DR (16, 18). As for melatonin delivery, intraperitoneal injection (31), oral administration (32), and intravitreal injection (33) were all used and tested the protective effects of melatonin on retinas in experimental animal models. In our study, considering the blood–ocular barrier and the possible side effects of systemic high-dose administration, we chose intravitreal injection to deliver melatonin directly into the eye. Based on the available literature, intravitreal melatonin (50–150 μg/kg body weight) did not induce retinal morphological changes in guinea pigs (34); even the high dose from 100 to 300 µg/kg body weight did not have any significant detrimental effects on the photoreceptor survival or visual function on mouse retina (33), indicating a wide safe dose range for intravitreal injection of melatonin. In a recent study, intravitreal delivery of melatonin (150–250 μg/kg body weight) could well maintain the thickness of the outer nuclear layer in the chemical-induced retinitis pigmentosa mouse model (35). In our study, we used Sprague-Dawley rats with the body weight about 150 g; the calculated dose range for intravitreal administration of melatonin was about 15~45 μg. Thus, we selected 30 μg for intravitreal injection and proved that melatonin treatment maintained the integrity of the iBRB and attenuated vascular leakage *via* directly protecting the endothelial cells (18). However, the optimal dose for intravitreal injection of melatonin merits further study.

In this study, we showed the evidence that activated microglia migrated to the outer retina and got a close interaction with the deep capillary plexus in diabetic rat retinas, and secreted several pro-inflammatory cytokines, resulting in the breakdown of iBRB. Melatonin suppressed the microglial overactivation and decreased its proliferation and migration in diabetic rat retinas, and thus decreased the capillary leakage and improved pericyte coverage. The *in vitro* study also confirmed the effect of melatonin, showing the decrease of pericyte apoptosis, the increase of barrier function, and the restoration of tight junctional proteins in endothelial cells caused by the supernatant from the activated microglia. Based on these results, we concluded that melatonin could protect iBRB by regulating microglial overactivation in DR.

In our previous study, melatonin alleviated vascular leakage in diabetic rat retina by direct suppression inflammatory cytokines *via* p38/TXNIP/NF-κB signaling pathways (18). Considering that activated microglia also release inflammatory cytokines under hypoxic conditions, we further investigated the effect of melatonin on microglial regulation and iBRB protection in DR. Consistent with our speculation, microglia produced abundant inflammatory cytokines such as TNF-α, iNOS, and IL-1β, as well as high levels of Arg-1 and CD206 (36). Melatonin regulated microglial activity by inhibiting inflammatory factor release and promoting anti-inflammatory factor secretion, further relieving the pericyte apoptosis and tight-junction loss. Although the involvement of hypoxia cannot be excluded, the present data suggested that activated microglia-derived inflammatory factors participated in iBRB disruption.

Based on bioinformatic analysis, the KEGG pathways of microglial activation were mostly enriched in PI3K/AKT, Stat3, and NF-κB signaling pathways, which led us to verify the changes of these pathways in activated microglia and the possible mechanisms of melatonin regulation. Consistently, the protein expressions of PI3K, p-Akt, p-Stat3, and p-NF-κB were increased in diabetic rat retinas and CoCl_2_-treated BV2 cells, which were reduced by melatonin treatment. With the corresponding inhibitors, PI3K/AKT/Stat3/NF-κB signaling pathways were verified in activated microglia, which were suppressed by melatonin.

A recent study found that the protective effects of melatonin mainly occur at the level of mitochondria relative to other cellular compartments, as melatonin synthetic enzymes have been identified in mitochondria (37). Under most conditions, the levels of reactive oxygen species (ROS) are delicately controlled by the mitochondrial antioxidant system and are maintained within a certain range suitable for serving as signaling molecules. However, excessive ROS levels under hyperglycemia result in oxidative stress, leading to cell and tissue damage. Indeed, melatonin is a potent direct free radical scavenger and also acts as an indirect antioxidant by enhancing the activities of antioxidant enzymes including mitochondrial superoxide dismutases (SODs), catalase, and glutathione reductase (38). Thus, substantially high melatonin is required for stabilizing the mitochondrial functions, especially in the antioxidant system (39).

In addition, glycolytic metabolism also increases in DR due to chronic hyperglycemia and hypoxia, which favors macrophage polarization from the anti-inflammatory type M2 to the pro-inflammatory type M1 (40). M1 macrophages secrete a variety of pro-inflammatory cytokines and chemokines, which causes excessive apoptosis in retina. Based on our preliminary results, we speculated that melatonin might promote microglial transformation from the pro-inflammatory to anti-inflammatory types through regulating energy metabolism and restoring ROS balance in mitochondria, which deserved further exploration.

**Figure 11:**
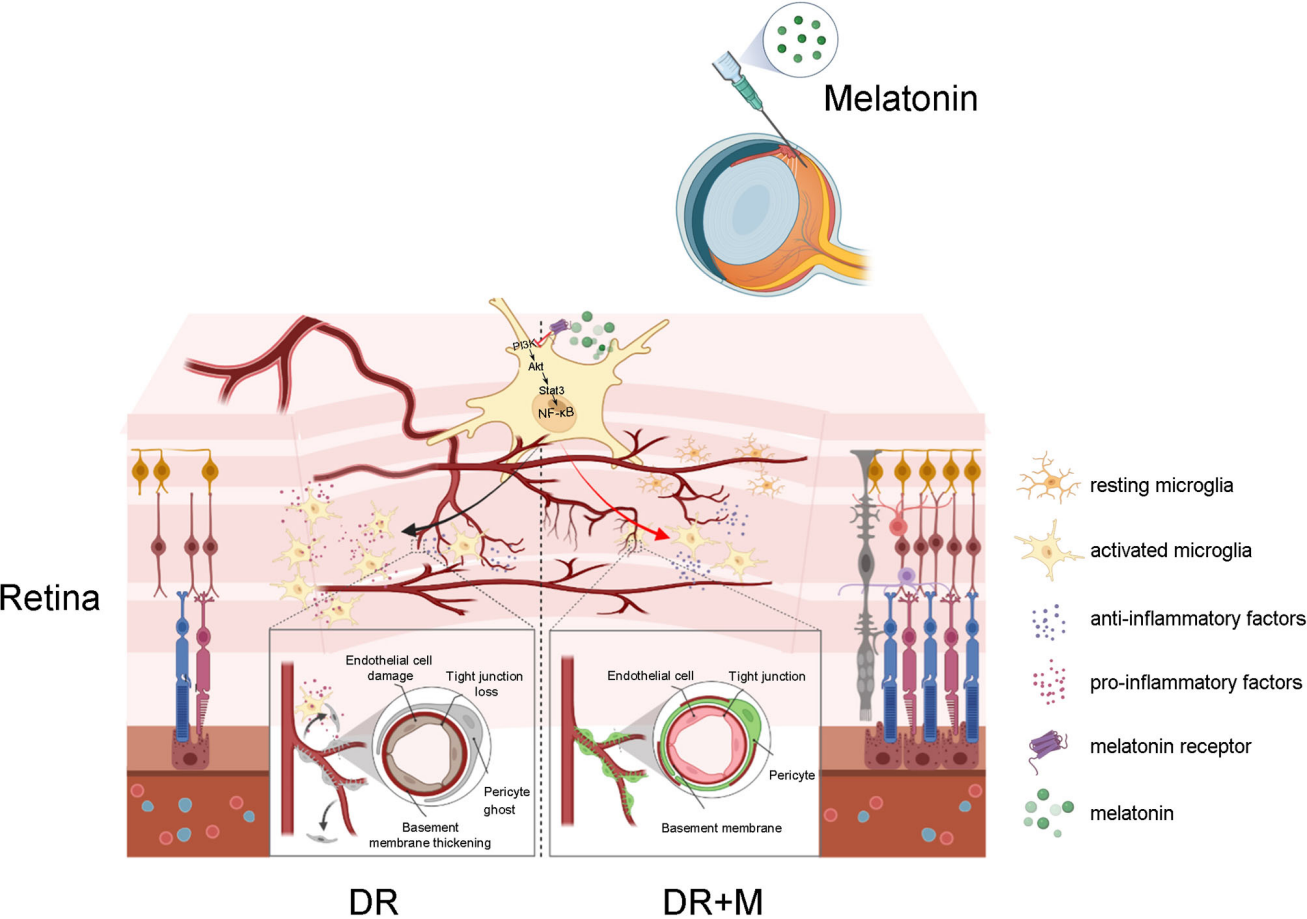
Schematic of the activation of microglia in diabetic retinopathy and the mechanism of melatonin to protect inner blood-retinal barrier by regulating microglia. DR, diabetic retinopathy; DR+M, diabetic retinopathy with melatonin treatment.

In conclusion, this study provides new insight into early events in the pathogenesis of DR, especially the contribution of activated microglia to iBRB breakdown and the protection of melatonin on iBRB by regulating microglia in DR (**Figure 11**). Evidence from both *in vitro* and *in vivo* experiments demonstrated that the pro-inflammatory factors released by activated microglia can cause the death of pericytes and endothelial cells, revealing the correlation between microglial activation and microvascular damage in DR. This study is also the first to provide evidence that melatonin protects pericytes and endothelial cells by deactivating microglia *via* inhibition of PI3K/Akt/Stat3/NF-κB signaling pathways, thereby preserving iBRB integrity.

## Data Availability Statement

Publicly available datasets were analyzed in this study. These data can be found here: https://www.ncbi.nlm.nih.gov/geo/query/acc.cgi?acc=GSE148350.

## Ethics Statement

All experiments were adhered to the ARVO Statement for the Use of Animals in Ophthalmic and Vision Research, and the protocols were approved by National Institutes of Health guide for the care and use of Laboratory animals (NIH Publications No. 8023) and the Committee on the Ethics of Animal Experiments of Tongji University (Permit Number: TJHBLAC-2021-06).

## Author Contributions

LT, G-TX, and JZ contributed to the conception and design of the experiments and were responsible for the data collection, analysis, and interpretation. CZ contributed to the data analysis and article revision. LT and JZ drafted the article. LL, HT, KL, DL, and QQ contributed to the data analysis and revised the article critically for important intellectual content. All authors approved the final version of the manuscript for submission. JZ and G-TX are guarantors of this work, who have full access to all the data in this study and take responsibility for the integrity and accuracy of the data. All authors contributed to the article and approved the submitted version.

## Funding

This study was supported by the National Natural Science Foundation of China (81870667, 81970810, 82171062), and grant from the Ministry of Science and Technology of China (2020YFA0113101).

## Conflict of Interest

The authors declare that the research was conducted in the absence of any commercial or financial relationships that could be construed as a potential conflict of interest.

## Publisher’s Note

All claims expressed in this article are solely those of the authors and do not necessarily represent those of their affiliated organizations, or those of the publisher, the editors and the reviewers. Any product that may be evaluated in this article, or claim that may be made by its manufacturer, is not guaranteed or endorsed by the publisher.
