# Supplementary material for: Melatonin Maintains Inner Blood–Retinal Barrier by Regulating Microglia via Inhibition of PI3K/Akt/Stat3/NF-κB Signaling Pathways in Experimental Diabetic Retinopathy
Source: Front Immunol. 2022 Mar 15;13:831660. doi: 10.3389/fimmu.2022.831660 (PMC8964465; doi:10.3389/fimmu.2022.831660)
Supplement: Supplementary file 1 [file Table_1.docx]

**Supplementary Materials**

**Table S1. Body weight and blood glucose level of normal and diabetic rats.**

|  | Baseline | | 8 weeks | |
| --- | --- | --- | --- | --- |
|  | Body weight  (g) | Blood glucose  (mmol/L) | Body weight  (g) | Blood glucose  (mmol/L) |
| Normal | 148.18 ± 1.98 | 8.54 ± 0.35 | 470.25 ± 4.41 | 8.08 ± 0.42 |
| Diabetic rats | 150.17 ± 1.45 | 27.16 ± 1.95* | 171.91 ± 4.35* | 29.66 ± 1.87* |

Note: All data were shown as mean ± SEM (n = 12). The difference for body weight or blood glucose level between normal group and diabetic rats was analyzed by Student’s t test. * indicated significant difference compared with normal group.

**Table S2. The information of endothelial labeling and the antibodies for immunofluorescence and Western blot.**

|  | Catalog number | Application | Vendor |
| --- | --- | --- | --- |
| Immunofluorescence |  |  |  |
| albumin | 66051 | 1:100 | Proteintech |
| CD16/32 | Ab223200 | 1:500 | Abcam |
| CD206 | 18704-1-AP | 1:500 | Proteintech |
| Claudin-5 | 35-2500 | 1:100 | Invitrogen |
| IBA-1 | 019-19741 | 1:500 | WAKO |
| IB4 | I21411 | 1:2,000 | Invitrogen |
| MT1 | sc-398788 | 1:50 | Santa Cruz |
| NG2 | 37-2700 | 1:500 | Invitrogen |
| ZO-1 | 61-7300 | 1:50 | Invitrogen |
| Rabbit IgG H&L (Alexa 555) | ab150078 | 1:500 | Abcam |
| Mouse IgG H&L (Alexa 488) | ab150117 | 1:500 | Abcam |
| Mouse IgG H&L (Alexa 647) | ab150115 | 1:500 | Abcam |
| Western blot | | | |
| Akt | 9252 | 1:1,000 | CST |
| phosphor-Akt | 9255 | 1:1,000 | CST |
| Arg-1 | 16001-1-AP | 1:10,000 | Proteintech |
| CD206 | 18704-1-AP | 1:10,000 | Proteintech |
| IBA-1 | ab178846 | 1:1,000 | Abcam |
| MT1 | sc-398788 | 1:200 | Santa Cruz |
| NF-κB | 8242 | 1:1,000 | CST |
| [phosphor-NF-κB](https://www.cst-c.com.cn/products/primary-antibodies/phospho-nf-kb-p65-ser536-93h1-rabbit-mab/3033?site-search-type=Products) | 3033 | 1:1,000 | CST |
| NG2 | 37-2700 | 1:1,000 | Invitrogen |
| PI3K | 4249T | 1:1,000 | CST |
| Stat3 | 8690 | 1:1,000 | CST |
| phosphor-Stat3 | 4511 | 1:1,000 | CST |
| HRP-conjugated β-actin | HRP-60008 | 1:5,000 | Proteintech |
| HRP anti-rabbit IgG | 15015 | 1:5,000 | Proteintech |
| HRP anti-mouse IgG | 15014 | 1:5,000 | Proteintech |

Akt (PKB), protein kinase B; Arg-1, arginase-1; CD16/32, cluster of differentiation 16/32; CD206, cluster of differentiation 206; IBA-1, ionized calcium binding adapter molecule 1; IB4, isolectin B4; MT1, melatonin receptor 1; NF-κB, nuclear factor κappa B; NG2, new glue 2; PI3K, phosphatidylinositol 3-kinase; Stat3, signal transducer and activator of transcription 3; ZO-1, zonula occludens 1.

**Table S3. The primer information**

| Arg-1 | forward 5′-CCTGAAGGAACTGAAAGGAAAG-3′  reverse 5′-TTGGCAGATATGCAGGGAGT-3′ |
| --- | --- |
| CCL-3 | forward 5′-AAGGATACAAGCAGCAGCGAGTA-3′  reverse 5′-TGCAGAGTGTCATGGTACAGAGAA-3′ |
| CCL-5 | forward 5′-CTGCTGCTTTGCCTACCTCT-3′  reverse 5′-CGAGTGACAAACACGACTGC-3′ |
| CXCL-10 | forward 5′-AAGGATACAAGCAGCAGCGAGTA-3′  reverse 5′-TGCAGAGTGTCATGGTACAGAGAA-3′ |
| CD206 | forward 5′-GTGGTCCTCCTGATTGTGATAG-3′  reverse 5′-CACTTGTTCCTGGACTCAGATTA-3′ |
| IL-1β | forward 5′-ATGCCACCTTTTGACAGTGATG-3′  reverse 5′-TGATGTGCTGCTGCGAGATT-3′ |
| iNOS | forward 5′-CCTGCTTTGTGCGAAGTGTC-3′  reverse 5′-CCCAAACACCAAGCTCATGC-3′ |
| MT1 | forward 5′-ATGGACCCCAACTGCTCCTG-3′  reverse 5′-AGGAGCAGCAGCTCTTCTTG-3′ |
| NG2 | forward 5′-GTCCTGCCTGTCAATGACCAAC-3′  reverse 5′-CGATGGTGTAGACCAGATCCTC-3′ |
| PDGFR-β | forward 5′-TGCAGACATCGAGTCCTCCAAC-3′  reverse 5′-GCTTAGCACTGGAGACTCGTTG-3′ |
| α-SMA | forward 5′-CTATGCCTCTGGACGCACAACT-3′  reverse 5′-CAGATCCAGACGCATGATGGCA-3′ |
| TGF-β | forward 5′-AGCTGCGCTTGCAGAGATTA-3′  reverse 5′-CACTTGTTCCTGGACTCAGATTA-3′ |
| TNF-α | forward 5′-ACGGCATGGATCTCAAAGAC-3′  reverse 5′-AGATAGCAAATCGGCTGACG-3′ |
| β-actin (human) | forward 5′-CACCATTGGCAATGAGCGGTTC-3′  reverse 5′-AGGTCTTTGCGGATGTCCACGT-3′ |
| β-actin (mouse) | forward 5′-AGGCGACAGCAGTTGGTTGGA-3′  reverse 5′-CACTTGTTCCTGGACTCAGATTA-3′ |

Arg-1, arginase-1; CCL-3, chemokine (C-C motif) ligand 3; CCL-5, chemokine (C-C motif) ligand 5; CD206, cluster of differentiation 206; CXCL-10, chemokine (C-X-C motif) ligand 10; IL-1β, interleukin-1β; iNOS, inducible nitric oxide synthase; MT1, melatonin receptor; NG2, new glue 2; PDGFR-β, platelet-derived growth factor-β; α-SMA, α-smooth muscle actin; TGF-β, transforming growth factor-β; TNF-α, tumor necrosis factor-α.
